# Understanding the Diversity of Pharmacotherapeutic Management of ADHD With Co-occurring Autism: An Australian Cross-Sectional Survey

**DOI:** 10.3389/fpsyt.2022.914668

**Published:** 2022-06-27

**Authors:** Olivia J. Mellahn, Rachael Knott, Jeggan Tiego, Kathryn Kallady, Katrina Williams, Mark A. Bellgrove, Beth P. Johnson

**Affiliations:** ^1^Faculty of Medicine, Nursing and Health Sciences, Turner Institute for Brain and Mental Health, Monash University, Melbourne, VIC, Australia; ^2^Department of Paediatrics, Paediatrics Education & Research, Monash University, Melbourne, VIC, Australia; ^3^Developmental Paediatrics, Monash Children's Hospital, Melbourne, VIC, Australia

**Keywords:** ADHD, attention, hyperactivity, impulsivity, methylphenidate, psychostimulant, autism, autism spectrum disorder

## Abstract

**Objectives:**

Attention deficit hyperactivity disorder (ADHD) frequently co-occurs with other neurodevelopmental diagnoses, such as autism spectrum disorder (autism), which can make clinical decision making around symptom management challenging for clinicians. There is a paucity of research examining pharmacotherapeutic management of children who have ADHD with co-occurring diagnoses. We aimed to report on the co-occurring diagnoses and symptom profile of children, and report on medication use, stratified by ADHD, autism and ADHD + autism diagnoses.

**Methods and Materials:**

Caregivers of 505 children (2–18 years) with ADHD (*n* = 239), autism (*n* = 117), and co-occurring ADHD + autism (*n* = 149) completed a questionnaire on current medication use and clinical rating scales about their child's symptoms, as part of a broader project investigating diagnosis and management of symptoms in children with ADHD or autism.

**Results:**

The parents of the ADHD group reported a higher proportion of their children had learning disorders (17.15%) and speech and language disorders (4.60%) compared to the parents of the autism and ADHD + autism groups. Parents of the ADHD + autism group reported higher proportions of intellectual disability (5.37%), oppositional defiant disorder (20.13%), anxiety (38.93%), depression (6.71%) and genetic conditions (3.36%) in their children, in comparison to the parents of the ADHD and autism groups. Children with ADHD were reported to be taking a higher proportion of psychotropic medication (90%), followed by ADHD + autism (86%) and autism (39%). The parents of children with ADHD + autism reported a higher proportion of non-stimulant ADHD medication (25.5%), antipsychotic (18.79%), antidepressant (22.15%) and melatonin (31.54%) use by their children, compared to the parents of the ADHD and autism groups.

**Conclusions:**

A similar proportion of children with ADHD + autism and ADHD were reported to be taking medication. However, the types of medication taken were different, as expected with reported co-occurring diagnoses. The complexity of symptoms and diagnoses in ADHD + autism warrants targeted research to optimize management and therapeutic outcomes.

## Introduction

Attention-deficit/hyperactivity disorder (ADHD) is a prevalent neurodevelopmental disorder, affecting approximately 5–7% of children (1, 2). ADHD is characterized by persistent inattention, and or hyperactivity and impulsivity (3). ADHD is a heterogeneous disorder, with a range of symptom and cognitive domains affected (4–7). The symptoms represent a broad spectrum of inattentive/disorganized, hyperactive and impulsive symptoms, such as difficulty following through on instructions, challenges completing schoolwork, and interrupting or intruding on others, which impact academic performance, social relationships and daily functioning. The heterogeneity within ADHD is evident from broad range of symptoms that individuals can present with, including inattention, hyperactivity and impulsivity (1, 8, 9).

Options for management of symptoms associated with ADHD encompass both psychotherapeutic and behavioral interventions (10–12). Stimulant medications, such as methylphenidate and dexamphetamine, have been front-line treatment options for decades for targeting hyperactivity and attentional difficulties (11–13). More recently, non-stimulant medications such as atomoxetine, clonidine and guanfacine have been utilized in conjunction with, or in place of, stimulant medications (12, 14, 15). This is consistent with current international guidelines that suggest that pharmacological therapy, when used in conjunction with behavioral and educational supports, is an effective treatment for ADHD (11).

In addition to within-disorder heterogeneity, ADHD often co-occurs with other conditions. Research shows that approximately 66% of children with ADHD have at least one co-occurring condition, including anxiety, depression and/or sleep conditions (16, 17). Autism spectrum disorder (autism) is a neurodevelopmental condition that often co-occurs with ADHD. Autism is characterized by deficits in social communication, and restrictive, repetitive and stereotyped patterns of behavior and interests (3). Autism affects approximately 1–3% of children (18, 19) and approximately 20–50% of children with ADHD present with autism symptoms, and 40–80% of children with autism present with ADHD symptoms (6, 20–22). Co-occurring ADHD and autism (hereafter ADHD + autism) is associated with greater symptom severity and complexity, which can make selecting the appropriate support or intervention for each child difficult for both clinicians and caregivers (4, 23, 24).

In contrast to ADHD, there are no pharmacological treatments that currently target the core social communication difficulties or repetitive behaviors of autism (25). However, antipsychotics, such as risperidone, are often prescribed to treat severe behavioral problems, such as aggression, in children with autism (25). Management of additional co-occurring conditions is also common for children with comorbid ADHD + autism, who are more likely to experience depression and/or anxiety, compared to children with ADHD or autism alone, and consequently may be prescribed antidepressants or anxiety reducing medications (26, 27). Thus, psychotropic medication use is more frequent in ADHD and ADHD + autism populations, in comparison to ASD alone (12, 28, 29). Clinical management of the breadth of ADHD symptom presentations and co-occurring conditions can therefore encompass stimulant and non-stimulant medications, antipsychotic and antidepressant medications, and medications for managing sleep behaviors, such as melatonin, alongside behavioral therapies (14, 15, 26, 30).

Sleep problems are prevalent in both children with ADHD and autism, and are particularly prevalent in children with ADHD + autism (31, 32), with melatonin often prescribed to assist with managing sleep behaviors (30, 33). Previous research has suggested that over 60% of children and adolescents with ADHD, autism, or ADHD + ASD experience sleep problems (31, 32). Efron et al. (30) found that 22% of ADHD children, recruited from pediatric clinics, were taking melatonin or clonidine for managing sleep problems. Identifying and managing sleep disturbance is important, as sleep problems have been associated with increased symptom severity in children with ADHD (30, 34). However, previous studies examining medication use in Australian populations have excluded melatonin as it is not considered a psychotropic medication (14, 15). Establishing the extent of melatonin use in children with ADHD and co-occurring diagnoses has thus far been challenging. This is due to the various ways in which parents are able to access melatonin. Within North America, melatonin is an over-the-counter medication, available without a prescription. Within Australia, melatonin has exclusively been available only via prescription until the start of 2022, however many parents access melatonin online without prescription. Thus, healthcare data relating to prescriptions does not fully capture the use of melatonin within ADHD populations in Australia.

Despite the high proportion of ADHD and co-occurring autism, there is little research on medication use in children with ADHD, autism and ADHD + autism. Factors that may be associated with different proportion of pharmacotherapy in these clinical populations include demographic variables (26, 34) and clinical variables, such as diagnosis and symptom severity, which can increase the likelihood of medication use (10, 12, 35). Previous studies have found that additional diagnoses were predictive of the amount of medication used in adolescents and adults with autism (36, 37). Coury et al. (38) compared children with autism to autism with co-occurring conditions, such as ADHD, bipolar disorder, depression and anxiety, and found that children with ASD and co-occurring diagnoses were more likely to be medicated. Rasmussen et al. (15) examined medication use in children and adolescents with autism in an Australian cohort. This study included a subgroup of children with autism and comorbid ADHD, however the sample was small (*n* = 32), and there was no sample of children with ADHD without comorbid autism.

A key limitation of previous studies of medication use relates to changes in diagnostic criteria that occurred with the introduction of DSM-5 in 2013 (3). DSM-5 allowed a dual diagnosis of ADHD + autism. Therefore, studies published prior to 2013 may not be representative of current clinical needs or treatment models. Moreover, past studies have omitted medications such as clonidine and melatonin, and the current extent of use of newer medications such as lisdexamfetamine and guanfacine has not been sufficiently captured (10, 12, 14, 15, 35). In order to understand the current state of medication use in children spanning ADHD, autism and ADHD + autism, it is necessary to investigate medication use in children diagnosed since the introduction of DSM-5, and to capture the full breadth of medications now available for symptom management.

To better characterize the medications currently used in symptom management among children with ADHD and co-occurring diagnoses, this study examines psychotropic medication use among children with ADHD, autism and ADHD + autism. This study included medications not previously surveyed in this context such as lisdexamfetamine, guanfacine, clonidine, and melatonin. Parents reported current medication use of their child and completed clinical rating scales to measure current symptom severity. The aims were: 1) to report on the co-occurring conditions and symptom profile of children, stratified by ADHD, autism and ADHD + autism diagnoses; and 2) to report on medication use of children with ADHD, autism and ADHD + autism.

## Methods and Materials

### Participants

**Figure 1:**
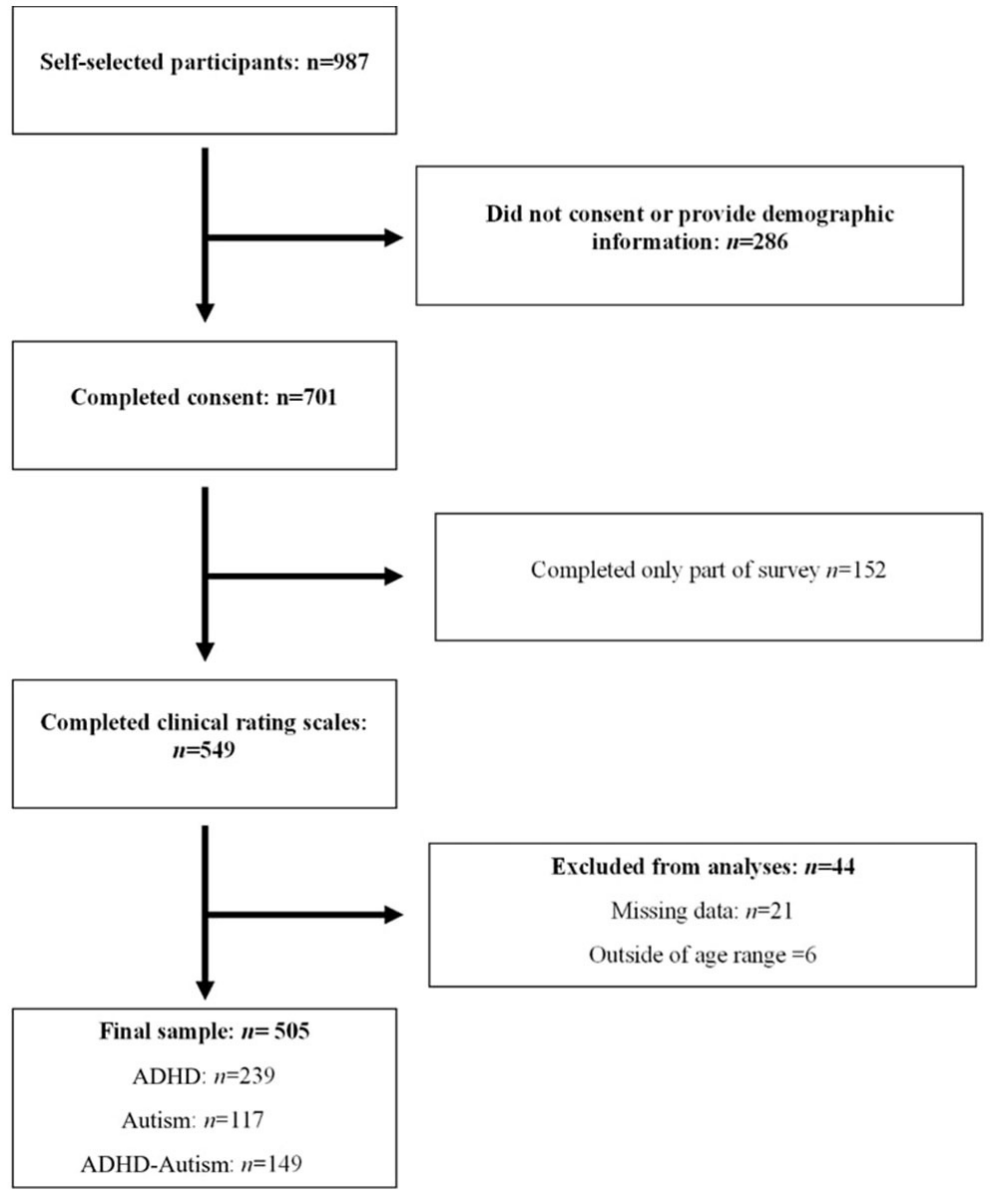
Participant inclusion and exclusion process.

Participants were the primary caregivers of 505 children (*M* = 9.64 years, *SD* = 3.12 years), diagnosed with ADHD (*n* = 239, male = 178), autism (*n* = 117, male = 80), or ADHD-autism (*n* = 149, male = 111) within the last 6 years. Participants were recruited using convenience sampling via physical and electronic flyers, advertising on social media, direct contact with schools, and via existing databases, see Figure 1. Parents reported the age at which a clinical diagnosis of autism and/or ADHD was made, by whom (type of clinician: pediatrician, psychiatrist, psychologist) and in which setting (e.g. public, private). Key inclusion criteria included a clinical diagnosis within the last 6 years to ensure the caregiver's recall was as accurate as possible.

A total of 701 families self-selected to complete consent and participate in the survey; 505 (72%) of whom completed the ASD and ADHD symptom rating scales between June 2019 and November 2020. Missing data for both the overall sample of 701 [Little's MCAR test, χ2 (55) = 44.88, *p* = 0.833] and the study sample of 505 [Little's MCAR test, χ2 (114) = 133.57, *p* = 0.102] was <5% and was missing completely at random (MCAR). In accordance with Little's MCAR test, a *p*-value of > 0.05 is interpreted as the data is missing completely at random. This indicated that analyses were unlikely to converge on biased estimates due to systematically missing values from some respondents related to extraneous variables, and missingness was ignorable (39).

### Measures

#### Diagnostic Process and Medication

The completed survey included questions about family demographics, the child's diagnostic journey, parental satisfaction and stress associated with the diagnostic process, and what interventions, supports and medications the child had previously and currently used. Data were collected to determine child age at the time of the survey, age at diagnosis, discipline area of the healthcare clinician who confirmed the diagnosis (e.g., pediatrician, psychiatrist or psychologist, multidisciplinary team) and healthcare setting (e.g., public or private). This information was repeated for each additional diagnosis (e.g. if autism was diagnosed in addition to ADHD). To minimize retrospective recall bias, only families who had received a diagnosis within the last 6 years were included in the dataset, excluding 6 children. To address the current aims of the study, examining co-occurring diagnoses and symptom profile of children diagnosed with ADHD, autism and ADHD + autism; and medication use in these populations, the scope of the present study was restricted specifically to information relating to diagnosis, current medication use, and current symptom severity only.

Data relating to other interventions, supports and diagnostic processes including time to diagnosis, and professionals involved in the diagnostic process will be reported elsewhere. The survey can be viewed: https://figshare.com/s/60d8d9f739fd459271c9.

The following are the medications included in analyses, listed by class, and were compiled from the Therapeutic Goods Administration (26). There were more medications queried within the survey, however only those with at least one participant taking them were included in analyses and are listed.

- *ADHD stimulants:*
  ◦ dexamphetamine;
  ◦ lisdexamfetamine (Vyvanse); and
  ◦ methylphenidate (Ritalin 10, Ritalin LA, Concerta Extended-Release Tablets).
- *ADHD non-stimulants:*
  ◦ atomoxetine (Strattera),
  ◦ clonidine (Catapres), and
  ◦ guanfacine (Intuniv).
- *Antipsychotics*: (all atypical):
  ◦ aripiprazole (Abilify),
  ◦ olanzapine,
  ◦ quetiapine (Seroquel), and
  ◦ risperidone (Risperdal).
- *Antidepressants:* (all selective serotonin reuptake inhibitors (SSRIs)):
  ◦ Citalopram (Cipramil),
  ◦ escitalopram (Lexapro),
  ◦ fluoxetine HCL (Prozac, Zactin tabs),
  ◦ fluvoxamine (Luvox, Voxam), and
  ◦ sertraline (Zoloft).
- *Antidepressants (other):*
  ◦ mirtazapine (Avanza).
- *Other:* Melatonin (Circadin).

This study included melatonin in the medication list as a large proportion of children with ADHD (30) and autism (33) have been shown to use melatonin to manage sleep behaviors; however, these are often not captured in studies of medication use in this population.

Part of this survey was constructed in line with a 2015 UK survey, with items added to better represent the National Disability Insurance Scheme (NDIS) system used in Australia for categorizing disorders and determining support (40).

#### Parent-Rated Symptom Rating Scales

Parents rated current autism and ADHD symptom severity via clinical rating scales. The Conners' Parent Rating Scale-Revised Long form (CPRS) was utilized to measure ADHD-related behaviors, and the Social Responsiveness Scale-2nd Edition (SRS-2) to measure autism-related behaviors.

##### Conners' Parent Rating Scale-Revised Long Form (CPRS)

The Conners' Parent Rating Scale-Revised Long form (CPRS) is an 80-item parent-report, with a 4-point Likert scale, measuring ADHD-related behaviors such as inattentive, hyperactive, anxious and impulsive behaviors (41). Age and gender matched *T*-scores were used for inattentive (Inattentive) subscale, and hyperactive-impulsive (Hyperactive) subscale. See Table 1 for the T-score descriptors (41, 42).

**Table 1:** CPRS:R-L and SRS-2 Severity Ratings.

**Measure**	***T*-score range**	**Description**
CPRS:R-L	≤ 59	Average/Within normal limits
	60–64	High Average
	65–69	Elevated
	≥ 70	Very Elevated
SRS-2	≤ 59	Within normal limits
	60–65	Mild
	66–75	Moderate
	≥ 76	Severe

##### Social Responsiveness Scale-2nd Edition (SRS-2)

The Social Responsiveness Scale-2nd Edition (SRS-2) is a 65-item parent-report measure with a 4-point Likert scale, that captures children's social and behavioral difficulties associated with autism (43). Gender matched *T*-scores were used for the DSM-5 social communication and interaction score (social communication index [SCI]), and the DSM-5 restrictive and repetitive behaviors (RRB) subscale, see Table 1 for the T-score descriptors (44).

##### Socio-Economic Status (SES)

Socio-economic status (SES) was grouped into low (lowest 25%), middle (middle 50%) and high (highest 25%) based on the postcode provided. SES was calculated from the index of relative socio-economic advantage and disadvantage (IRSAD) calculated from the 2016 census data, where postcodes are assigned a percentile based on their relative advantage or disadvantage (45, 46).

##### Polypharmacy

Polypharmacy was defined as concurrent use of two or more individual medications, in accordance with a 2017 systematic review that found this was the most common definition of polypharmacy in studies examining psychotropic medication use in autism (35). We also included melatonin within this list of medications, as up to 20% of children with ADHD, autism or ADHD + autism have been reported to take melatonin (30–32). Although melatonin is not a psychotropic medication, it is an additional medication that is use to manage sleep behaviors, which are often reported as contributing to behavioral management of ADHD and/or autism.

#### Procedures

The survey was administered using Research Electronic Data Capture (REDCap) hosted and managed by Helix [Monash University, (47, 48)]. REDCap is a secure, web-based software platform designed to support data capture for research studies, providing 1) an intuitive interface for validated data capture; 2) audit trails for tracking data manipulation and export procedures; 3) automated export procedures for seamless data downloads to common statistical packages; and 4) procedures for data integration and interoperability with external sources. One parent and or a caregiver completed the online questionnaire about the child. Participants were informed that each questionnaire was for one child only.

We sought to determine whether parental reported diagnoses of ADHD, autism and ADHD + autism were corroborated by symptom ratings collected at the time of survey on both the CPRS Inattentive and Hyperactive scales, and the SRS RRB and SCI scales. Significant group differences in the expected directions were observed, such that the ADHD and ADHD + autism groups had significantly elevated scores on the CPRS Inattentive and Hyperactive scales, while the autism and ADHD + autism groups showed significantly elevated scores on SRS RRB and SCI scales (see S1 for significance testing).

### Statistical Analyses

Descriptive statistics including frequencies were reported for co-occurring diagnoses and symptom profile stratified by ADHD, autism and ADHD + autism diagnosis. Frequencies were reported for medication use stratified by ADHD, autism and ADHD + autism diagnosis as well as biological sex. All calculations were performed using SPSS 26 and Microsoft Excel 2019.

We sought to determine whether parental reported diagnoses of ADHD, autism and ADHD-autism were supported by symptom ratings collected at the time of survey on both the CPRS Inattentive and Hyperactive scales, and the SRS RRB and SCI scales. Section 1 of the Supplementary Material contains means and standard deviations of group symptom severity, in addition to a Kruskal-Wallis ANOVA for each symptom scale and pairwise comparisons of diagnosis.

## Results

### Clinical and Socio-Demographic Characteristics

Table 2 displays demographic and clinical data as a function of diagnostic group.

**Table 2:** Clinical and socio-demographic characteristics.

		**ADHD**	**Autism**	**ADHD + Autism**	**Total**
		**Count (%)**	**Count (%)**	**Count (%)**	**Count (%)**
	Total	239 (47.33%)	117 (23.17%)	149 (29.5%)	505 (100%)
Sex	Male	178 (74.48%)	80 (68.38%)	111 (74.5%)	369 (73.07%)
	Female	61 (25.52%)	37 (31.62%)	38 (25.5%)	136 (26.93%)
Age group (years)	≤ 5 years	10 (4.18%)	32 (27.35%)	5 (3.36%)	47 (9.31%)
	6–10 years	143 (59.83%)	52 (44.44%)	100 (67.11%)	295 (58.42%)
	11–15 years	78 (32.64%)	27 (23.08%)	41 (27.52%)	146 (28.91%)
	≥ 16 years	8 (3.35%)	6 (5.13%)	3 (2.01%)	17 (3.37%)
Additional diagnosis		121 (50.63%)	52 (44.44%)	80 (53.69%)	253 (50.1%)
	Intellectual disability	2 (0.84%)	6 (5.13%)	8 (5.37%)	16 (3.17%)
	Epilepsy	4 (1.67%)	0 (0.00%)	2 (1.34%)	6 (1.19%)
	Learning disorder	41 (17.15%)	2 (1.71%)	12 (8.05%)	55 (10.89%)
	Oppositional defiant disorder	40 (16.74%)	3 (2.56%)	30 (20.13%)	73 (14.46%)
	Anxiety	59 (24.69%)	30 (25.64%)	58 (38.93%)	147 (29.11%)
	Depression	9 (3.77%)	2 (1.71%)	10 (6.71%)	21 (4.16%)
	Speech or language disorder	11 (4.60%)	5 (4.27%)	3 (2.01%)	19 (3.76%)
	Genetic	2 (0.84%)	2 (1.71%)	5 (3.36%)	9 (1.78%)
	Other	45 (18.83%)	25 (21.37%)	25 (16.78%)	95 (18.81%)
Socioeconomic status	Low	25 (10.46%)	23 (19.66%)	29 (19.46%)	77 (15.25%)
	Middle	117 (48.95%)	56 (47.86%)	77 (51.68%)	250 (49.5%)
	High	97 (40.59%)	38 (32.48%)	43 (28.86%)	178 (35.25%)
SRS-2 DSM RRB descriptors	Within normal limits	61 (25.52%)	3 (2.56%)	7 (4.7%)	71 (14.06%)
	Mild	36 (15.06%)	4 (3.42%)	4 (2.68%)	44 (8.71%)
	Moderate	76 (31.8%)	32 (27.35%)	32 (21.48%)	140 (27.72%)
	Severe	66 (27.62%)	78 (66.67%)	106 (71.14%)	250 (49.5%)
SRS-2 DSM SCI descriptors	Within normal limits	76 (31.8%)	6 (5.13%)	7 (4.7%)	89 (17.62%)
	Mild	49 (20.5%)	6 (5.13%)	6 (4.03%)	61 (12.08%)
	Moderate	67 (28.03%)	28 (23.93%)	46 (30.87%)	141 (27.92%)
	Severe	47 (19.67%)	77 (65.81%)	90 (60.4%)	214 (42.38%)
Conners' DSM Inattentive descriptors	Within normal limits	21 (8.79%)	20 (17.09%)	10 (6.71%)	51 (10.1%)
	High Average	19 (7.95%)	12 (10.26%)	16 (10.74%)	47 (9.31%)
	Elevated	34 (14.23%)	24 (20.51%)	25 (16.78%)	83 (16.44%)
	Very Elevated	165 (69.04%)	61 (52.14%)	98 (65.77%)	324 (64.16%)
Conners' DSM Hyperactive descriptors	Within normal limits	35 (14.64%)	21 (17.95%)	16 (10.74%)	72 (14.26%)
	High Average	14 (5.86%)	13 (11.11%)	3 (2.01%)	30 (5.94%)
	Elevated	21 (8.79%)	18 (15.38%)	15 (10.07%)	54 (10.69%)
	Very Elevated	169 (70.71%)	65 (55.56%)	115 (77.18%)	349 (69.11%)

*The total row and column were calculated with respect to the total sample N = 505. All other rows were calculated with respect to n for each diagnostic group, e.g. n = 184 for ADHD group, n = 94 for autism group and n = 124 for the ADHD + autism group. CPRS, Conners' Parent Rating Scale-Revised Long form; Inattentive, subscale; Hyperactive, Hyperactive-impulsive subscale; SRS-2, Social Responsiveness Scale-2nd Edition; RRB, restrictive and repetitive behaviors; SCI, social communication index*.

The final sample included 505 children, of which 47% (239) had a parent-reported ADHD diagnosis, 23% (117) an autism diagnosis and 29.5% (149) had a co-occurring ADHD + autism diagnosis. When including the children who had a co-occurring ADHD + autism diagnosis in the ADHD group, 77% of the children had an ADHD diagnosis, with 39% of children with ADHD also having an autism diagnosis.

#### Age Range

The majority of children across the ADHD (59%), autism (44%) and ADHD + autism groups (67%) were in the 6–10 years age range, however a large percentage (27%) of the autism sample were under 5 years age, compared to the ADHD (4%) and ADHD + autism (3%) group.

#### Co-occurring Diagnoses

When stratifying by group, 50% of the ADHD group reported additional co-occurring diagnoses, while 44% of the autism group and 53% of the ADHD + autism group reported additional diagnoses.

The ADHD group had the lowest rate of reported intellectual disability (0.84%), compared to the autism (5%) and the comorbid ADHD + autism (2%) groups. In contrast, the ADHD group reported the highest rate of learning disorders 17%, followed by the ADHD + autism group at 8% and the autism group 2%. Similarly, the ADHD group reported the most speech and language disorders (4%) compared to the autism (4%) and the ADHD + autism group (2%).

Caregivers of the ADHD + autism group reported the highest proportion of co-occurring oppositional defiant disorder (ODD, 20%) in their children, followed by the ADHD group (16%) and the autism group (2%). Additionally, caregivers of the ADHD + autism group reported the highest rates of anxiety (38%) and depression (6%) in their children. The autism group had the highest proportion of other conditions (21%), including conditions as different as congenital heart disease to verbal dyspraxia.

#### Socio-Economic Status

The socio-economic status (SES) varied little across the ADHD, autism and ADHD + autism groups and with 49%, 48 and 52% across the middle SES, respectively.

#### ADHD and Autism Symptoms

**Figure 2:**
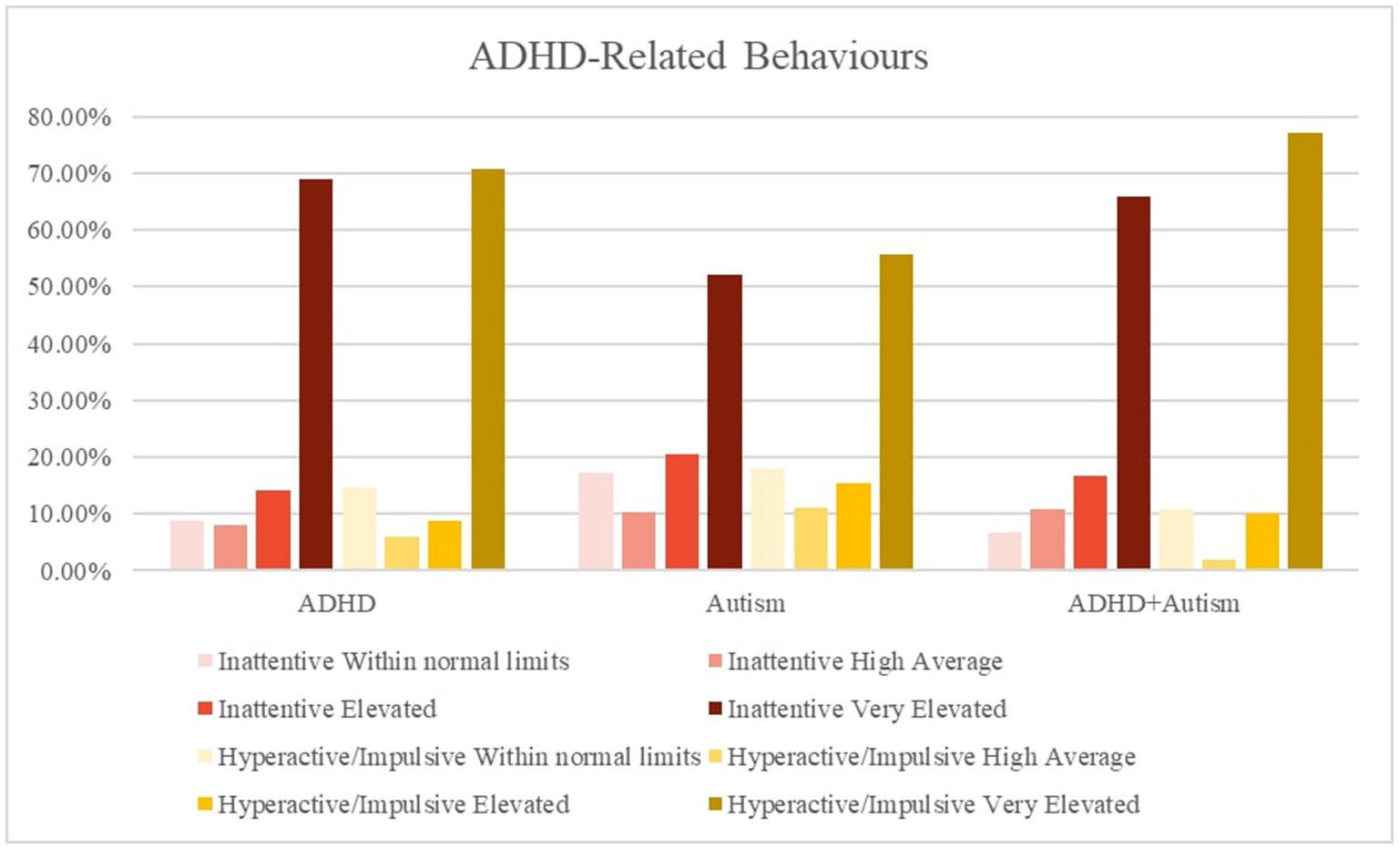
Graphical summary of the ADHD symptoms stratified by diagnosis and hyperactive/impulsive and inattention symptoms. The CPRS:R-L descriptors relate to the standardized scoring as follows, ≤ 59 = *Average/Within normal limits*, 60–64 = *High Average*, 65–69 = *Elevated*, 70 ≥ = *Very Elevated*. CPRS = Conners' Parent Rating Scale-Revised Long form. Inattentive, subscale; Hyperactive, Hyperactive-impulsive subscale; SRS-2, Social Responsiveness Scale-2nd Edition; RRB, restrictive and repetitive behaviors; SCI, social communication index.

**Figure 3:**
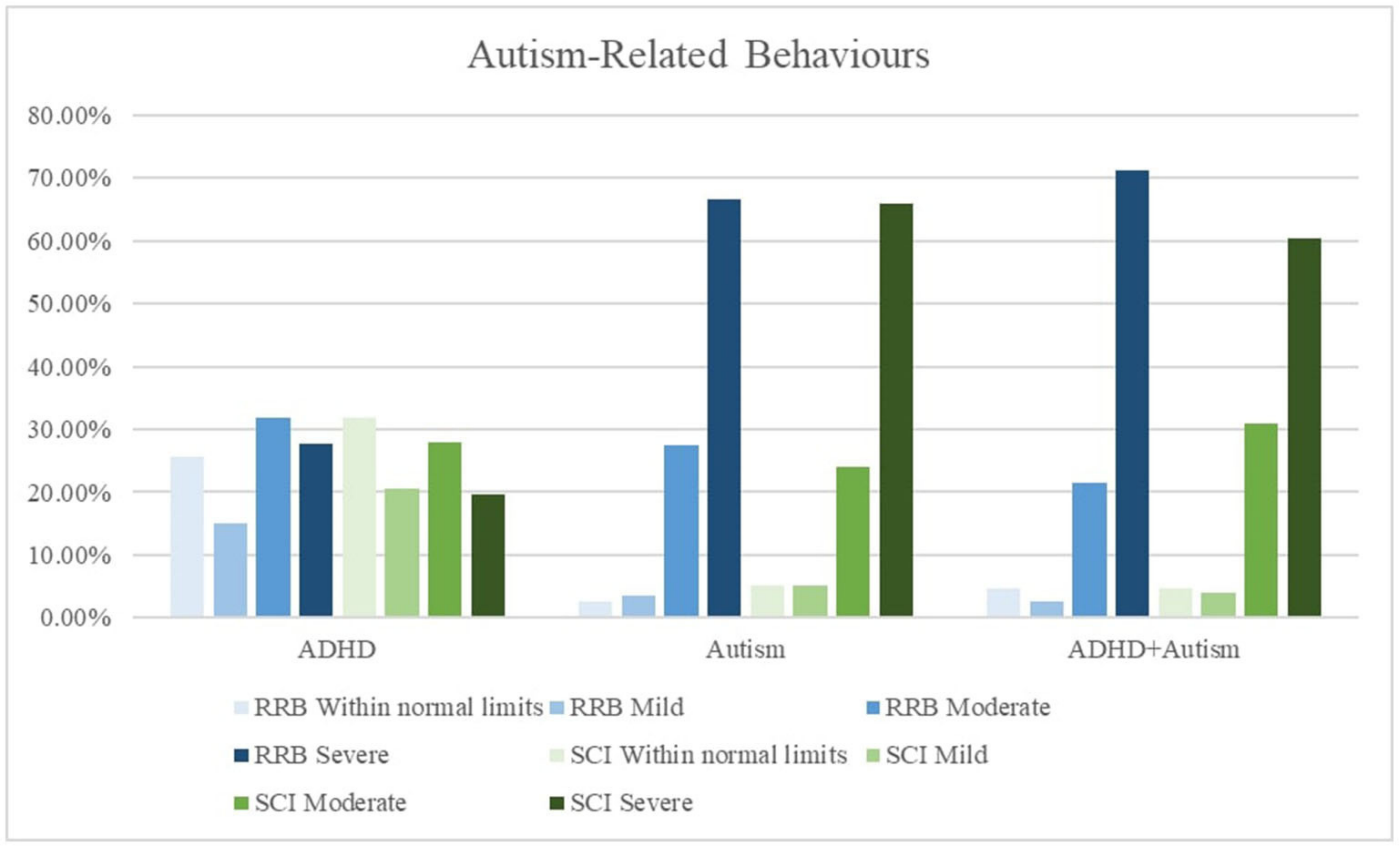
Graphical summary of the autism symptoms stratified by diagnosis and restrictive repetitive behaviors (RRB) and social communication issues (SCI). The SRS-2 descriptors relate to the standardized scoring as follows, ≤ 59 = *Within normal limits*, 60–65 = *Mild*, 66–75 = *Moderate*, 76 ≥ = *Severe*. CPRS, Conners' Parent Rating Scale-Revised Long form. Inattentive, subscale. Hyperactive, Hyperactive-impulsive subscale; SRS-2, Social Responsiveness Scale-2nd Edition; RRB, restrictive and repetitive behaviors; SCI, social communication index.

Caregivers of all three groups reported high levels of inattention and hyperactive symptoms in their children. As expected, there were less frequent reports by the autism group (52, 56%) of very elevated inattention and hyperactive symptoms compared to the ADHD (69, 70%) and the ADHD + autism group (66, 77%). The autism (67, 66%) and ADHD + autism (71, 60%) groups had high levels of severe restrictive and repetitive behaviors (RRB) and social communication and interaction (SCI) behaviors, while the ADHD group did not. Figures 2, 3 displays frequency of the symptom severity for the ADHD- and autism-related behaviors, stratified by group.

### Medication Use Stratified by Diagnostic Group and sex

Table 3 displays current medication use within medication classes, stratified by diagnosis and sex (see Supplementary Table S2 for stratification by age). Overall, 77% of the total sample were taking at least one medication. Specifically, 90% of the ADHD sample, 39% of the autism sample and 86% of the ADHD + autism sample were taking at least one medication. Additionally, the ADHD + autism group (52%) also reported the highest proportion of polypharmacy (concurrent use of two or more medications, including melatonin), compared to the ADHD (21%) and the autism (22%) groups. The most frequently reported medication classes for the total sample were stimulant medications (59%), followed by melatonin (24%), non-stimulant ADHD medications (21%), antidepressants (11%) and antipsychotics (8%).

**Table 3:** Medication use stratified by diagnostic group and sex.

	**ADHD (*n =* 239)**			**Autism (*n =* 117)**			**ADHD + Autism (*n =* 149)**			**Total (*n =* 505)**		
	**Male**	**Female**	**Total**	**Male**	**Female**	**Total**	**Male**	**Female**	**Total**	**Male**	**Female**	**Total**
	**Count (%)**	**Count (%)**	**Count (%)**	**Count (%)**	**Count (%)**	**Count (%)**	**Count (%)**	**Count (%)**	**Count (%)**	**Count (%)**	**Count (%)**	**Count (%)**
Currently using medication ≥1	**162 (67.78%)**	**52 (21.76%)**	**214 (89.54%)**	**25 (21.37%)**	**20 (17.09%)**	**45 (38.46%)**	**97 (65.1 %)**	**31 (20.81%)**	**128 (85.91%)**	**284 (56.24%)**	**103 (20.4 %)**	**387 (76.63%)**
**Stimulants total**	**146 (61.09%)**	**47 (19.67%)**	**193 (80.75%)**	**3 (2.56%)**	**2 (1.71%)**	**5 (4.27%)**	**77 (51.68%)**	**21 (14.09%)**	**98 (65.77%)**	**226 (44.75%)**	**70 (13.86%)**	**296 (58.61%)**
Ritalin 10 (Methylphenidate)	51 (21.34%)	17 (7.11%)	68 (28.45%)	1 (0.85%)	1 (0.85%)	2 (1.71%)	19 (12.75%)	5 (3.36%)	24 (16.11%)	71 (14.06%)	23 (4.55%)	94 (18.61%)
Ritalin LA (Methylphenidate)	31 (12.97%)	10 (4.18%)	41 (17.15%)	0 (0.00%)	0 (0.00%)	0 (0.00%)	15 (10.07%)	6 (4.03%)	21 (14.09%)	46 (9.11%)	16 (3.17%)	62 (12.28%)
Vyvanse (Lisdexamfetamine)	36 (15.06%)	11 (4.60%)	47 (19.67%)	0 (0.00%)	0 (0.00%)	0 (0.00%)	23 (15.44%)	6 (4.03%)	29 (19.46%)	59 (11.68%)	17 (3.37%)	76 (15.05%)
Concerta ER (Methylphenidate)	37 (15.48%)	13 (5.44%)	50 (20.92%)	0 (0.00%)	0 (0.00%)	0 (0.00%)	23 (15.44%)	6 (4.03%)	29 (19.46%)	60 (11.88%)	19 (3.76%)	79 (15.64%)
Dexamfetamine	4 (1.67%)	2 (0.84%)	6 (2.51%)	2 (1.71%)	1 (0.85%)	3 (2.56%)	3 (2.010%)	2 (1.34%)	5 (3.36%)	9 (1.78%)	5 (0.99%)	14 (2.77%)
**Non-stimulant total**	**42 (17.57%)**	**13 (5.44%)**	**55 (23.01%)**	**10 (8.55%)**	**3 (2.56%)**	**13 (11.11%)**	**30 (20.13%)**	**8 (5.37%)**	**38 (25.5%)**	**82 (16.24%)**	**24 (4.75%)**	**106 (20.99%)**
Clonidine	19 (7.95%)	9 (3.77%)	28 (11.72%)	10 (8.55%)	3 (2.56%)	13 (11.11%)	14 (9.40%)	4 (2.68%)	18 (12.08%)	43 (8.51%)	16 (3.17%)	59 (11.68%)
Atomoxetine	8 (3.35%)	2 (0.84%)	10 (4.18%)	0 (0.00%)	0 (0.00%)	0 (0.00%)	5 (3.36%)	2 (1.34%)	7 (4.70%)	13 (2.57%)	4 (0.79%)	17 (3.37%)
Guanfacine	17 (7.11%)	2 (0.84%)	19 (7.95%)	1 (0.85%)	0 (0.00%)	1 (0.85%)	13 (8.72%)	2 (1.34%)	15 (10.07%)	31 (6.14%)	4 (0.79%)	35 (6.93%)
**Antipsychotics total**	**4 (1.67%)**	**1 (0.42%)**	**5 (2.09%)**	**6 (5.13%)**	**3 (2.56%)**	**9 (7.69%)**	**23 (15.44%)**	**5 (3.36%)**	**28 (18.79%)**	**33 (6.53%)**	**9 (1.78%)**	**42 (8.32%)**
Risperidone	4 (1.67%)	0 (0.00%)	4 (1.67%)	5 (4.27%)	3 (2.56%)	8 (6.84%)	19 (12.75%)	4 (2.68%)	23 (15.44%)	28 (5.54%)	7 (1.39%)	35 (6.93%)
Aripiprazole	0 (0.00%)	0 (0.00%)	0 (0.00%)	1 (0.85%)	0 (0.00%)	1 (0.85%)	2 (1.34%)	1 (0.67%)	3 (2.01%)	3 (0.59%)	1 (0.20%)	4 (0.79%)
Olanzapine	0 (0.00%)	0 (0.00%)	0 (0.00%)	0 (0.00%)	0 (0.00%)	0 (0.00%)	1 (0.67%)	0 (0.00%)	1 (0.67%)	1 (0.20%)	0 (0.00%)	1 (0.20%)
Quetiapine	0 (0.00%)	1 (0.42%)	1 (0.42%)	0 (0.00%)	0 (0.00%)	0 (0.00%)	1 (0.67%)	0 (0.00%)	1 (0.67%)	1 (0.20%)	1 (0.20%)	2 (0.40%)
**Antidepressants total**	**7 (2.93%)**	**4 (1.67%)**	**11 (4.6%)**	**2 (1.71%)**	**11 (9.4%)**	**13 (11.11%)**	**20 (13.42%)**	**13 (8.72%)**	**33 (22.15%)**	**29 (5.74%)**	**28 (5.54%)**	**57 (11.29%)**
Citalopram	0 (0.00%)	0 (0.00%)	0 (0.00%)	0 (0.00%)	0 (0.00%)	0 (0.00%)	1 (0.67%)	0 (0.00%)	1 (0.67%)	1 (0.20%)	0 (0.00%)	1 (0.20%)
Escitalopram	1 (0.42%)	0 (0.00%)	1 (0.42%)	0 (0.00%)	1 (0.85%)	1 (0.85%)	2 (1.34%)	1 (0.67%)	3 (2.01%)	3 (0.59%)	2 (0.40%)	5 (0.99%)
Fluoxetine HCl	4 (1.67%)	4 (1.67%)	8 (3.35%)	2 (1.71%)	6 (5.13%)	8 (6.84%)	11 (7.38%)	6 (4.03%)	17 (11.41%)	17 (3.37%)	16 (3.17%)	33 (6.53%)
Fluvoxamine	0 (0.00%)	0 (0.00%)	0 (0.00%)	0 (0.00%)	1 (0.85%)	1 (0.85%)	2 (1.34%)	1 (0.67%)	3 (2.01%)	2 (0.40%)	2 (0.40%)	4 (0.79%)
Mirtazapine	1 (0.42%)	0 (0.00%)	1 (0.42%)	0 (0.00%)	0 (0.00%)	0 (0.00%)	1 (0.67%)	0 (0.00%)	1 (0.67%)	2 (0.40%)	0 (0.00%)	2 (0.40%)
Sertraline	1 (0.42%)	0 (0.00%)	1 (0.42%)	0 (0.00%)	3 (2.56%)	3 (2.56%)	3 (2.01%)	5 (3.36%)	8 (5.37%)	4 (0.79%)	8 (1.58%)	12 (2.38%)
**Melatonin**	**33 (13.81%)**	**16 (6.69%)**	**49 (20.5%)**	**14 (11.97%)**	**12 (10.26%)**	**26 (22.22%)**	**37 (24.83%)**	**10 (6.71%)**	**47 (31.54%)**	**84 (16.63%)**	**38 (7.52%)**	**122 (24.16%)**
Polypharmacy ≥2 including melatonin	69 (28.87%)	23 (9.62%)	92 (38.49%)	11 (9.40%)	10 (8.55%)	21 (17.95%)	57 (38.26%)	20 (13.42%)	77 (51.68%)	137 (27.13%)	53 (10.50%)	190 (37.62%)

*Percentages were calculated with respect to total n within each diagnostic group, e.g. n = 239 for ADHD group, N = 505 for the total sample. Polypharmacy ≥2 including melatonin, concurrent use of two or more medications, including melatonin*.

*Bold values indicate the medication class totals*.

Stimulant use was most frequently reported in the ADHD group, with 81% taking at least one stimulant medication, with methylphenidate being the reported stimulant (46%) taken. The ADHD + autism group reported less stimulant use (66%) while the autism group reported least frequent use again (4%). The most frequently reported stimulant medications in the ADHD + autism group were lisdexamfetamine (19%) and methylphenidate (extended release, 19%).

The ADHD + autism group had the highest use of non-stimulant, antipsychotic, antidepressant and melatonin classes. The ADHD and ADHD+autism groups had similar frequency of use of non-stimulant ADHD medication (26%, 23%) with less frequent use in the ASD group (11%). Clonidine was the most used non-stimulant across all three groups, followed by Guanfacine. The ADHD + autism group reported the most use of antipsychotics (19%) and antidepressants (22%), followed by the autism group (8, 11%) and the ADHD group (2, 5%). Risperidone was the most reported antipsychotic and fluoxetine the most reported antidepressant by all three groups.

All three groups reported frequent use of melatonin, with the ADHD + autism group reporting the highest use (32%), followed by the autism group (22%) and then the ADHD group (21%).

## Discussion

The range of pharmacotherapeutic options for treatment and support of ADHD has expanded over the past decade, as has our understanding of the complex presentation of ADHD. Co-occurring diagnosis of ADHD and autism can represent a significant challenge for clinicians in deciding appropriate pharmacological therapies for individuals. The purpose of this study was to better understand the range of co-occurring diagnoses and symptoms and pharmacological management for ADHD, autism and ADHD + autism presentations.

### Co-occurring Diagnoses and Symptom Complexity

The first aim of this study was to understand the range of co-occurring diagnoses and the symptom profiles of children stratified by ADHD, autism and ADHD + autism diagnoses, to capture the breadth of complexity in symptom presentation when considering pharmacotherapeutic treatment options.

In the present study, over a third of children with ADHD had a co-occurring autism diagnosis. Children with co-occurring ADHD + autism are more likely to present with a broader range of clinical symptoms, and greater symptom severity (4, 23, 24, 49). They also have greater impairments in adaptive functioning compared to children with either ADHD or autism, and are more likely to have a delayed autism diagnosis which can act as a barrier to accessing early intervention (50–52). As expected, the ADHD group reported more inattentive and hyperactive symptoms than autism symptoms, and the autism group reported more restrictive and repetitive behaviors (RRB) and social communication and interaction (SCI) symptoms, compared to inattentive and hyperactive symptoms. Consistent with previous reports (49) the ADHD + autism group reported high levels of both ADHD and autism symptoms, and reported the highest proportion of severe RRBs and very elevated hyperactive symptoms.

#### Co-occurring Conditions

This study found that 54% of children with ADHD + autism reported at least one additional co-occurring condition, compared to 51% of ADHD children and 44% of autistic children. The ADHD + autism group reported higher proportions of intellectual disability, ODD, anxiety, depression and genetic conditions, which is consistent with previous findings (7, 49, 53–55). The ADHD group reported the highest proportion of learning disorders, and speech and language disorders, with prevalence rates consistent with other previously reported data (56).

Previous research reported that children with autism (57%) were more likely to have at least one co-occurring condition, compared to children with ADHD (40%, 57). A possible explanation is that Steensel et al. (57) had a smaller sample size, with diagnosis made against DSM-IV criteria. In contrast, this project recruited children who had received their diagnosis since the DSM-5 was released in 2013. Our findings are broadly consistent with more recent data that suggests individuals with ADHD, autism and ADHD + autism display more specific patterns of co-occurring conditions. For example, a 2019 study examined patterns of psychiatric co-occurring conditions and found those with ADHD showed higher prevalence of mood conditions compared to those with autism who display higher prevalence of schizophrenia, while those with ADHD + autism displaying a higher prevalence compared to both ADHD and autism (58). An alternate explanation for difference is that we have explicitly included a co-occurring ADHD-autism group in our analyses.

In terms of support and therapeutic options, learning disorders and speech and language disorders are often supported by speech pathology, and educational remediation and intervention (59–61), however the focus of this research is on pharmacotherapies, therefore co-occurring conditions managed by medication will be the focus of this discussion.

#### Anxiety, Depression, and Oppositional Defiant Disorder

Our findings show that children with ADHD + autism had higher prevalence of anxiety (39%) and depression (7%), compared to the autism only group (26, 2%, respectively) and the ADHD group (25, 4%, respectively). The ADHD (17%) and the ADHD+autism (20%) group also reported higher prevalence of ODD compared to the autism (3%) group. In Icelandic children with ADHD, 19% of the children met cut-off criteria for ODD, 42% for anxiety, and 21% for depression (62). A UK study in adults with autism reported high prevalence of concurrent anxiety (27%) and depression (23%) (63). A 2019 meta-analysis found that individuals with autism were most likely to have a co-occurring ADHD diagnosis (28%) followed by anxiety (20%), sleep-wake disorders (13%), ODD and conduct disorders (11%) and mood conditions (9%). Overall, the prevalence of anxiety and depression for children with autism and ADHD + autism reported within this study mirrors that found in Rasmussen et al. (15) Australian study; however our findings are lower compared to international findings. A previous Australian study of boys with autism found higher prevalence of anxiety (43%) and depression (9%, 64). The elevated levels of anxiety reported by Bitsika et al. (64) relative to the present study may reflect anxiety captured by symptom rating scale cut-offs, rather than parent reported diagnosis.

### Medication Use

Greater symptom severity and complexity can make selecting the appropriate support or intervention for each child difficult for both clinicians and caregivers (4, 23, 24). Current guidelines for both ADHD and autism advise treating each co-occurring condition based on the individual diagnostic treatment guidelines. However, this can pose a problem for clinicians as many of the symptoms can between diagnoses (23, 65). Therefore, to better understand current treatment practices, the second aim of this study sought to capture the range of pharmacotherapies used.

#### Prevalence of Medication Use

Children with ADHD (90%) and children with ADHD+autism (86%) had higher prevalence of reported medication use, compared to children with autism (39%). Children with ADHD+autism predominantly reported non-stimulant ADHD medication, antipsychotic, antidepressant, and melatonin use. Parents of children with ADHD + autism reported a higher proportion of polypharmacy (defined here as the concurrent use of two or more medications, including melatonin), compared to children With ADHD or autism.

Similar to our study, in North America 82% of children diagnosed with ADHD and 25–40% of those with autism were prescribed psychotropic medications (28). This is in accordance with previous Australian studies, where 78% of children with ADHD + autism were provided one or more psychotropic medications, whereas only 35% of children with autism were prescribed medication (15).

Our findings for current medication use are slightly higher than previous reports from North America (12, 38), Australia (15), and the United Kingdom (66). One source of difference in medication use lies in our inclusion of medications such as lisdexamfetamine, guanfacine, clonidine and melatonin for the first time. Another possible difference is that children with ADHD + autism also had higher prevalence of anxiety and depression compared to the autism group and the ADHD group as reported above, which may explain higher medication use in this group. Lastly, a possible difference is the definition of polypharmacy; the most commonly used definition is concurrent use of two or more individual medications (36), however previous studies may have used alternative definitions.

#### Types of Medication Use Across ADHD, Autism, and Co-occurring ADHD + Autism

The most commonly used medications in the ADHD group were the stimulant and non-stimulant ADHD medications, which is consistent with current international treatment guidelines that suggest stimulant therapy as one of the first-line treatments for ADHD in children and adolescents (11, 25). The most used medications in the autism group were melatonin and antidepressants. This is expected as antidepressants have been reported as the most prescribed medication for individuals with autism (15, 67, 68) and we found high prevalence of depression and anxiety in children with autism (34%, see Table 1). The ADHD + autism group had the highest use in every medication class except for stimulants, with the most reported medications in this group being stimulants and melatonin. This is consistent with previous research that found children with ADHD + autism had higher use of stimulant medications, when compared with autistic children (15).

Overall, a quarter of the sample were taking melatonin. Previous research has found that sleep problems are prevalent in children with ADHD (61%), autism (65%) and ADHD + autism (66%) (31, 32). In an Australian study, it was reported 22% of ADHD children were taking melatonin or clonidine for managing sleep problems, which is consistent with our results (30). We found that the ADHD + autism (32%) group had reported higher melatonin use compared to the ADHD (21%) and autism (22%) groups. Previous findings have found that children with ADHD + autism experienced similar levels of sleep disturbance as children with ADHD (31, 69). Screening and treating for sleep disturbance early is important, as sleep problems have been associated with increased symptom severity in children with ADHD (30, 70).

### Strengths and Limitations

The reliance of our study on parental report is both a strength and a limitation. Previous studies have utilized or merged parental reports with healthcare data, examining prescription and dispensing of medications by health professionals, often resulting in higher sample sizes and more objective data (12, 15, 26). However, public healthcare data for prescription and dispensing of medications may not capture prevalence of actual usage, for example for preparations available without a prescription like melatonin. In addition, parental report facilitated the use of clinical rating scales to measure current autism and ADHD symptom severity. Future research utilizing both parent report and healthcare data would assist in validating parent-reported diagnoses and medication use.

Our study had more survey completions by parents of children with ADHD and ADHD + autism than parents of children with autism. This is likely due to the sampling strategy used, where parents self-selected to participate in the study. Our findings may be non-representative, however the description of medication and polypharmacy use within our cohort is in keeping with other reports and adds to information indicating the complexity of co-occurring ADHD and autism, and the way they are managed (71).

## Conclusion

Overall, the ADHD + autism group reported the highest proportion of comorbidities, including intellectual disability, oppositional defiant disorder, anxiety and depression. The severity of ADHD and autism symptoms was as expected for diagnoses reported by parents. Medication use overall was highest in children with ADHD, however, polypharmacy was highest in children with ADHD + autism.

Our findings are similar to others reporting the prevalence of medication use, with new knowledge about children diagnosed with ADHD + autism. The complexity of ADHD and autism presentations and their co-occurrence should be the subject of future research to investigate the efficacy of the medications used, in a way that allows clinicians, parents and young people to make decision about first line and additional medication that is suited to their symptoms, needs and priorities.

## Data Availability Statement

The raw data supporting the conclusions of this article will be made available by the authors, without undue reservation.

## Ethics Statement

The studies involving human participants were reviewed and approved by Monash University Human Research Ethics Committee. The patients/participants provided their written informed consent to participate in this study.

## Author Contributions

OM conceptualized and designed the study, designed the data collection instruments, collected data, carried out the analyses, drafted the initial manuscript, and reviewed and revised the manuscript for important intellectual content. RK conceptualized and designed the study, designed the data collection instruments, collected data, and reviewed and revised the manuscript for important intellectual content. JT and KW contributed to the analyses and interpretation of data and critically reviewed the manuscript for important intellectual content. KK conceptualized and designed the study and reviewed and revised the manuscript for important intellectual content. MB and BJ conceptualized and designed the study, coordinated and supervised data collection, and critically reviewed the manuscript for important intellectual content. All authors approved the final manuscript as submitted and agree to be accountable for all aspects of the work.

## Funding

This work is supported by a grant from the Medical Research Future Fund (MRFF) to Prof. Bellgrove and Dr. Johnson, EPCD000002. Prof. Bellgrove is supported by a Senior Research Fellowship from the National Health and Medical Research Council (NHMRC) of Australia, 1154378. BJ is supported by a Peter Doherty Fellowship from the NHMRC APP1112348. JT was supported by NHMRC Project Grants 1002458 and 1046054. The NHMRC and MRFF had no role in the design and conduct of the study.

## Conflict of Interest

The authors declare that the research was conducted in the absence of any commercial or financial relationships that could be construed as a potential conflict of interest.

## Publisher's Note

All claims expressed in this article are solely those of the authors and do not necessarily represent those of their affiliated organizations, or those of the publisher, the editors and the reviewers. Any product that may be evaluated in this article, or claim that may be made by its manufacturer, is not guaranteed or endorsed by the publisher.
