# Supplementary material for: Understanding the Diversity of Pharmacotherapeutic Management of ADHD With Co-occurring Autism: An Australian Cross-Sectional Survey
Source: Front Psychiatry. 2022 Jun 27;13:914668. doi: 10.3389/fpsyt.2022.914668 (PMC9271966; doi:10.3389/fpsyt.2022.914668)
Supplement: Supplementary file 1 [file Table_1.DOCX]

Supplementary Material

Contents

[1 Supplementary Information: Group Differences in Symptom Severity 2](#_Toc97818773)

[1.1 Figure S1.1. Inattentive symptoms 3](#_Toc97818774)

[1.2 Figure S1.2. Hyperactive/Impulsive symptoms 4](#_Toc97818775)

[1.3 Figure S1.3. Restrictive and repetitive behaviour (RRB) symptoms 5](#_Toc97818776)

[1.4 Figure S1.4. Social communication (SCI) symptoms 6](#_Toc97818777)

[2 Supplementary Information: Medication Use by Age Group 8](#_Toc97818778)

# Supplementary Information: Group Differences in Symptom Severity

The ADHD group had significantly elevated scores on the CPRS Inattentive [*M*_autism_ = 70, *M*_ADHD_ = 74] and CPRS Hyperactive [*M*_autism_ = 71, *M*_ADHD_ = 76] compared to the autism group, whereas the autism group had significantly elevated scores on SRS RRB [*M*_autism_ = 80, *M*_ADHD_ = 68] and SRS SCI [*M*_autism_ = 78, *M*_ADHD_ = 66] compared the ADHD group. Although the autism group did have high CPRS Inattentive and CPRS Hyperactive scores, they were significantly lower than the ADHD Group (see Table S1.1 and S1.2). The ADHD+autism group were not significantly different from the autism group for SRS RRB [*M*_ADHD+autism_ = 80] and SRS SCI [*M*_ADHD+autism_ =78] but were rated significantly higher on these measures than the ADHD group. The ADHD+autism group were not significantly different to the ADHD group on CPRS Inattentive [*M*_ADHD+autism_ =74] or CPRS Hyperactive measures [*M*_ADHD+autism_ =78] but did differ from the autism group on these same measures.

**Table S1.1 Means and Standard Deviations of Group Symptom Severity**

|  | ADHD | | Autism | | ADHD-Autism | |
| --- | --- | --- | --- | --- | --- | --- |
|  | M | SD | M | SD | M | SD |
| CPRS Inattentive | 74.059 | 10.330 | 70.333 | 11.384 | 74.174 | 9.950 |
| CPRS Hyperactive | 75.607 | 13.014 | 70.812 | 12.204 | 77.537 | 11.086 |
| SRS RRB | 68.423 | 12.195 | 79.709 | 9.954 | 80.315 | 9.680 |
| SRS SCI | 65.732 | 11.265 | 78.043 | 8.981 | 78.181 | 9.176 |

*Note:* CPRS = Conners’ Parent Rating Scale-Revised Long form. Inattentive = DSM-IV aligned inattentive subscale. Hyperactive = DSM-IV aligned hyperactive and or impulsive subscale. SRS = Social Responsiveness Scale, 2^nd^ Edition (SRS-2). RRB = DSM-5 restrictive and repetitive behaviours subscale. SCI = DSM-5 social communication index.


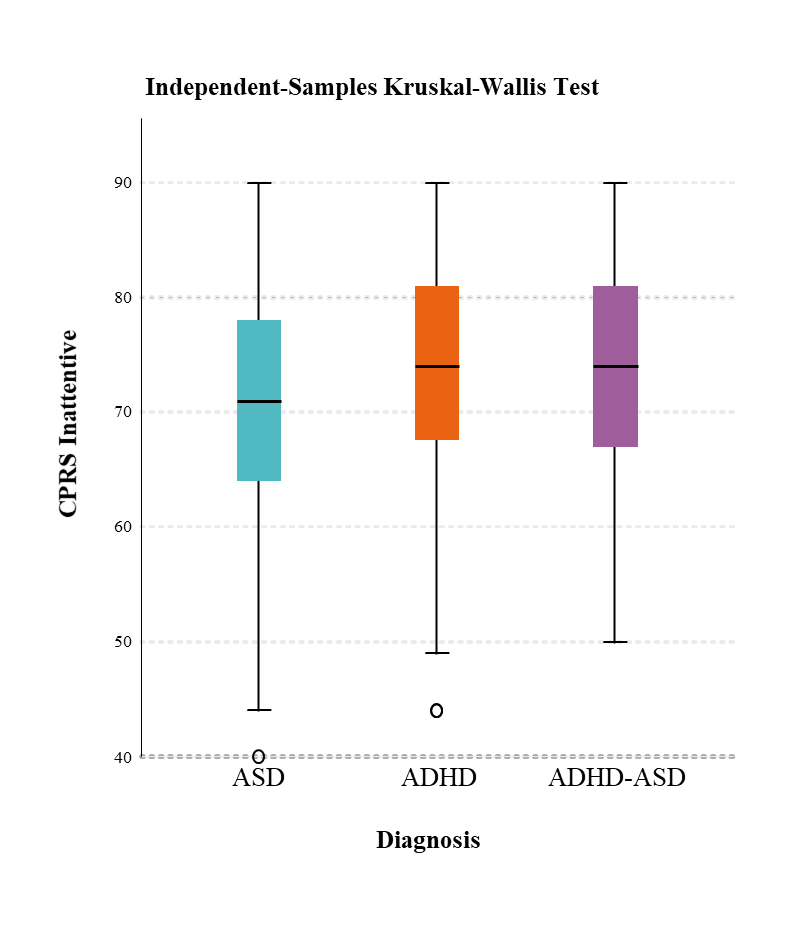


## *Figure S1.1.* Inattentive symptoms

Boxplot for the inattentive symptom scale stratified by group.

A Kruskal-Wallis ANOVA indicated that there were statistically significant differences of large effect between the Inattentive symptoms reported for autism, ADHD and ADHD-autism children, *χ*^2^=11.13, *df*=2, *N*=505, *p*<.01, with a small effect size, Cohen’s *f*=.15. Table S1.1 displayed the means and standard deviations by diagnostic group, and Table S1.2 displays the pairwise comparisons.


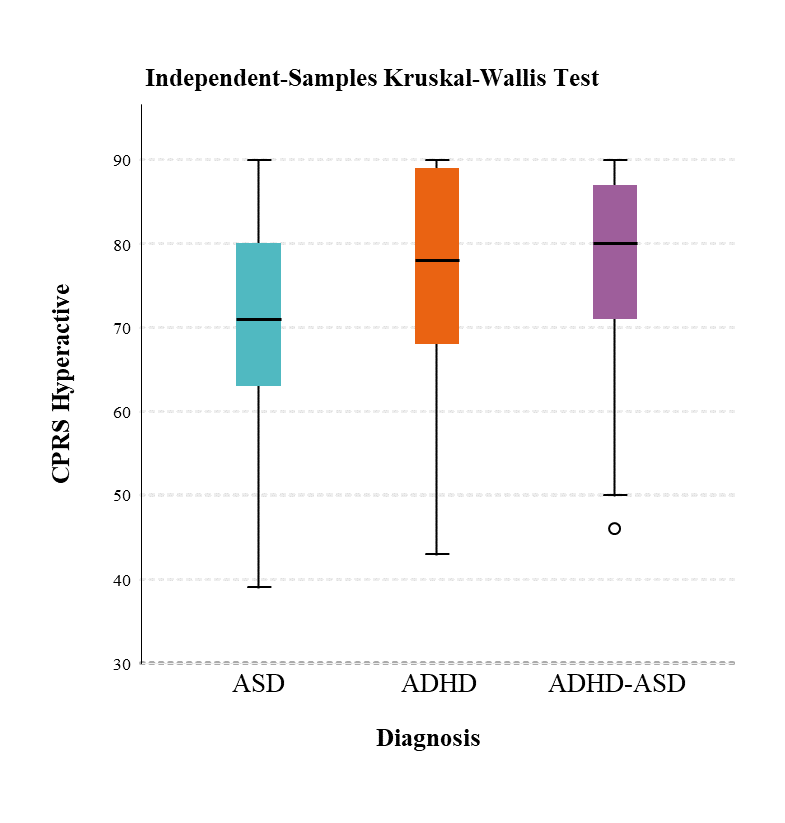


## *Figure S1.2.* Hyperactive/Impulsive symptoms

Boxplot for the hyperactive symptom scale stratified by group.

A Kruskal-Wallis ANOVA indicated that there were statistically significant differences of large effect between the Hyperactive symptoms reported for autism, ADHD and ADHD-autism children, *χ*^2^=20.79, *df*=2, *N*=505, *p*<.001, with a small effect size, Cohen’s *f*=.21. Table S1.1 displayed the means and standard deviations by diagnostic group, and Table S1.2 displays the pairwise comparisons.


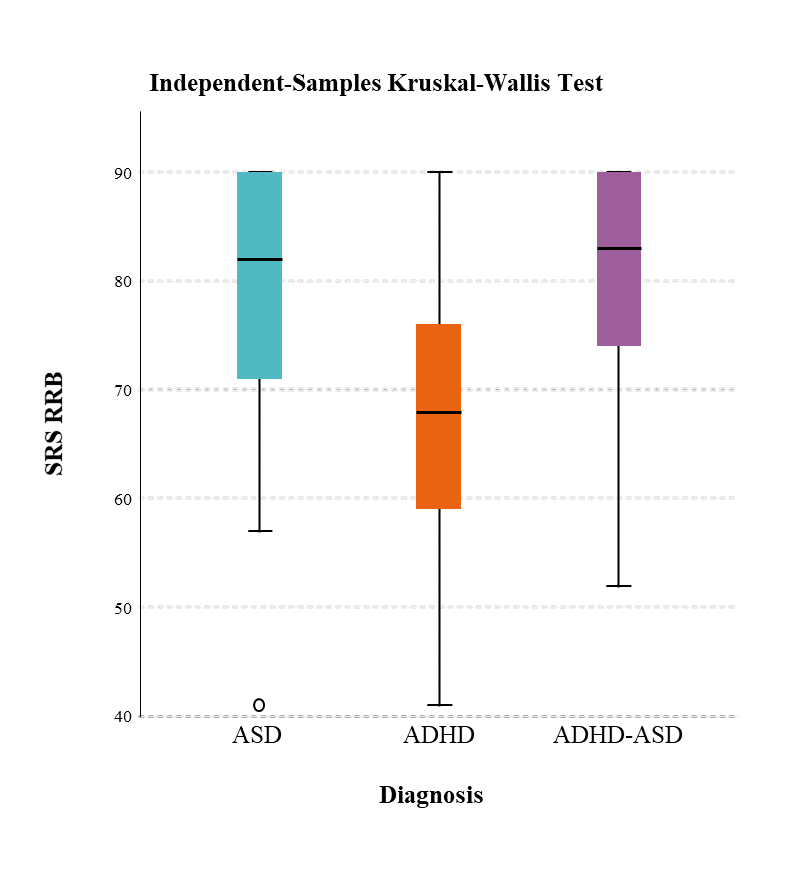


## *Figure S1.3*. Restrictive and repetitive behaviour (RRB) symptoms

Boxplot for the RRB symptom scale stratified by group.

A Kruskal-Wallis ANOVA indicated that there were statistically significant differences of large effect between the RRB symptoms reported for autism, ADHD and ADHD-autism children, *χ*^2^=109.96, *df*=2, *N*=505, *p*<.001, with a large effect size, Cohen’s *f*=.53. Table S1.1 displayed the means and standard deviations by diagnostic group, and Table S1.2 displays the pairwise comparisons.


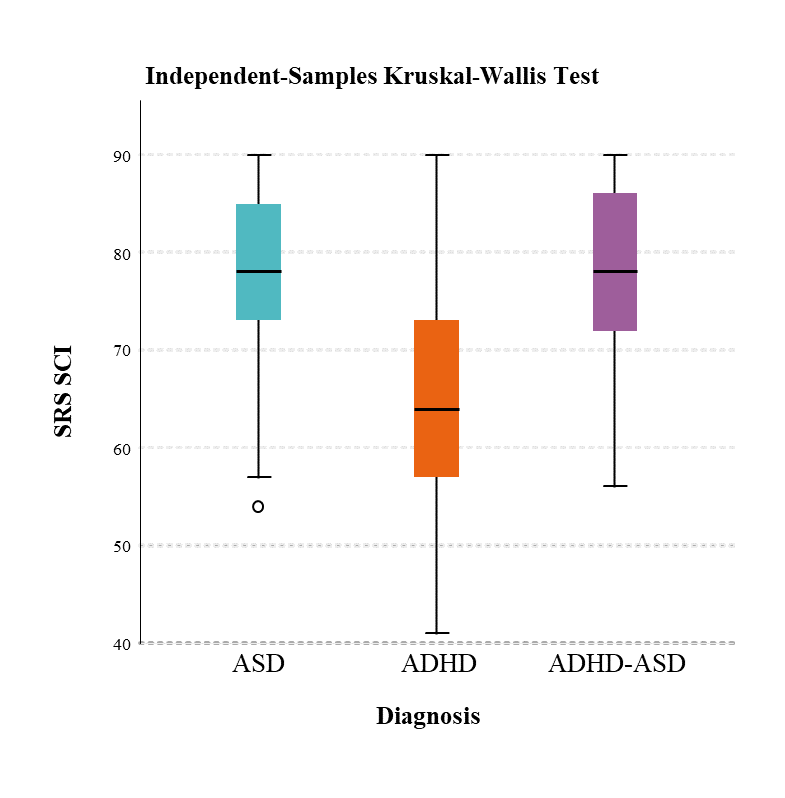


## *Figure S1.4.* Social communication (SCI) symptoms

Boxplot for the SCI symptom scale stratified by group.

A Kruskal-Wallis ANOVA indicated that there were statistically significant differences of large effect between the SCI symptoms reported for autism, ADHD and ADHD-autism children, *χ*^2^=135.82, *df*=2, *N*=505, *p*<.001, with a large effect size, Cohen’s *f*=.61. Table S1.1 displayed the means and standard deviations by diagnostic group, and Table S1.2 displays the pairwise comparisons.

**Table S1.2 Pairwise Comparisons of Diagnosis**

| **Sample 1-Sample 2** | **Test Statistic** | **Std. Error** | **Std. Test Statistic** | **Sig. ^a^** | **Adj. Sig.^b^** |
| --- | --- | --- | --- | --- | --- |
| **CPRS Inattentive** | | | | | |
| Autism - ADHD-Autism | -50.497 | 18.003 | -2.805 | 0.005 | 0.015 |
| Autism - ADHD | -51.755 | 16.444 | -3.147 | 0.002 | 0.005 |
| ADHD-Autism - ADHD | 1.258 | 15.213 | 0.083 | 0.934 | 1.000 |
| **CPRS Hyperactive** | | | | | |
| Autism - ADHD | -60.427 | 16.413 | -3.682 | 0.000 | 0.001 |
| Autism - ADHD-Autism | -78.749 | 17.968 | -4.383 | 0.000 | 0.000 |
| ADHD - ADHD-Autism | -18.322 | 15.184 | -1.207 | 0.228 | 0.683 |
| **SRS RRB** | | | | | |
| ADHD - Autism | 131.331 | 16.401 | 8.007 | 0.000 | 0.000 |
| ADHD - ADHD-Autism | -139.185 | 15.173 | -9.173 | 0.000 | 0.000 |
| Autism - ADHD-Autism | -7.854 | 17.955 | -0.437 | 0.662 | 1.000 |
| **SRS SCI** | | | | | |
| ADHD - Autism | 151.328 | 16.450 | 9.199 | 0.000 | 0.000 |
| ADHD - ADHD-Autism | -151.512 | 15.218 | -9.956 | 0.000 | 0.000 |
| Autism - ADHD-Autism | -0.184 | 18.009 | -0.010 | 0.992 | 1.000 |
| *Note:* Each row tests the null hypothesis that the Sample 1 and Sample 2 distributions are the same.  *a.* Asymptotic significances (2-sided tests) are displayed. The significance level is .05. | | | | | |
| *b.* Significance values have been adjusted by the Bonferroni correction for multiple tests. | | |  |  |  |

# Supplementary Information: Medication Use by Age Group

**Table S2**

*Medication use stratified by diagnosis and age*

|  | ADHD (*n*=239) | | | | Autism (*n*=117) | | | | ADHD-Autism (*n*=149) | | | | Total sample (*N*=505) | | | |
| --- | --- | --- | --- | --- | --- | --- | --- | --- | --- | --- | --- | --- | --- | --- | --- | --- |
|  | <6 | 6-10 | 11-15 | 16+ | <6 | 6-10 | 11-15 | 16+ | <6 | 6-10 | 11-15 | 16+ | <6 | 6-10 | 11-15 | 16+ |
| **Currently using ≥1** | 7 (2.93%) | 131 (54.81%) | 68 (28.45%) | 8 (3.35%) | 7 (5.98%) | 26 (22.22%) | 9 (7.69%) | 3 (2.56%) | 3 (2.01%) | 86 (57.72%) | 36 (24.16%) | 3 (2.01%) | 17 (3.37%) | 243 (48.12%) | 113 (22.38%) | 14 (2.77%) |
| **Stimulants total** | 5 (2.09%) | 117 (48.95%) | 64 (26.78%) | 7 (2.93%) | 1 (0.85%) | 1 (0.85%) | 2 (1.71%) | 1 (0.85%) | 2 (1.34%) | 69 (46.31%) | 25 (16.78%) | 2 (1.34%) | 8 (1.58%) | 187 (37.03%) | 91 (18.02%) | 10 (1.98%) |
| Ritalin 10 (Methylphenidate) | 3 (1.26%) | 46 (19.25%) | 19 (7.95%) | 0 (0%) | 0 (0%) | 0 (0%) | 1 (0.85%) | 1 (0.85%) | 0 (0%) | 14 (9.4%) | 9 (6.04%) | 1 (0.67%) | 3 (0.59%) | 60 (11.88%) | 29 (5.74%) | 2 (0.4%) |
| Ritalin LA (Methylphenidate) | 0 (0%) | 27 (11.3%) | 13 (5.44%) | 1 (0.42%) | 0 (0%) | 0 (0%) | 0 (0%) | 0 (0%) | 0 (0%) | 18 (12.08%) | 3 (2.01%) | 0 (0%) | 0 (0%) | 45 (8.91%) | 16 (3.17%) | 1 (0.2%) |
| Vyvanse (Lisdexamfetamine) | 0 (0%) | 24 (10.04%) | 21 (8.79%) | 2 (0.84%) | 0 (0%) | 0 (0%) | 0 (0%) | 0 (0%) | 1 (0.67%) | 23 (15.44%) | 4 (2.68%) | 1 (0.67%) | 1 (0.2%) | 47 (9.31%) | 25 (4.95%) | 3 (0.59%) |
| Concerta ER (Methylphenidate) | 0 (0%) | 28 (11.72%) | 20 (8.37%) | 2 (0.84%) | 0 (0%) | 0 (0%) | 0 (0%) | 0 (0%) | 0 (0%) | 17 (11.41%) | 12 (8.05%) | 0 (0%) | 0 (0%) | 45 (8.91%) | 32 (6.34%) | 2 (0.4%) |
| Dexamfetamine | 2 (0.84%) | 2 (0.84%) | 0 (0%) | 2 (0.84%) | 1 (0.85%) | 1 (0.85%) | 1 (0.85%) | 0 (0%) | 1 (0.67%) | 2 (1.34%) | 2 (1.34%) | 0 (0%) | 4 (0.79%) | 5 (0.99%) | 3 (0.59%) | 2 (0.4%) |
| **Non-stimulant total** | 3 (1.26%) | 34 (14.23%) | 17 (7.11%) | 1 (0.42%) | 4 (3.42%) | 8 (6.84%) | 1 (0.85%) | 0 (0%) | 0 (0%) | 26 (17.45%) | 12 (8.05%) | 0 (0%) | 7 (1.39%) | 68 (13.47%) | 30 (5.94%) | 1 (0.2%) |
| Clonidine | 1 (0.42%) | 18 (7.53%) | 9 (3.77%) | 0 (0%) | 4 (3.42%) | 8 (6.84%) | 1 (0.85%) | 0 (0%) | 0 (0%) | 14 (9.4%) | 4 (2.68%) | 0 (0%) | 5 (0.99%) | 40 (7.92%) | 14 (2.77%) | 0 (0%) |
| Atomoxetine | 0 (0%) | 4 (1.67%) | 5 (2.09%) | 1 (0.42%) | 0 (0%) | 0 (0%) | 0 (0%) | 0 (0%) | 0 (0%) | 4 (2.68%) | 3 (2.01%) | 0 (0%) | 0 (0%) | 8 (1.58%) | 8 (1.58%) | 1 (0.2%) |
| Guanfacine | 2 (0.84%) | 12 (5.02%) | 5 (2.09%) | 0 (0%) | 0 (0%) | 1 (0.85%) | 0 (0%) | 0 (0%) | 0 (0%) | 10 (6.71%) | 5 (3.36%) | 0 (0%) | 2 (0.4%) | 23 (4.55%) | 10 (1.98%) | 0 (0%) |
| **Antipsychotics total** | 0 (0%) | 4 (1.67%) | 0 (0%) | 1 (0.42%) | 0 (0%) | 7 (5.98%) | 2 (1.71%) | 0 (0%) | 1 (0.67%) | 16 (10.74%) | 11 (7.38%) | 0 (0%) | 1 (0.2%) | 27 (5.35%) | 13 (2.57%) | 1 (0.2%) |
| Risperidone | 0 (0%) | 4 (1.67%) | 0 (0%) | 0 (0%) | 0 (0%) | 7 (5.98%) | 1 (0.85%) | 0 (0%) | 1 (0.67%) | 14 (9.4%) | 8 (5.37%) | 0 (0%) | 1 (0.2%) | 25 (4.95%) | 9 (1.78%) | 0 (0%) |
| Aripiprazole | 0 (0%) | 0 (0%) | 0 (0%) | 0 (0%) | 0 (0%) | 0 (0%) | 1 (0.85%) | 0 (0%) | 0 (0%) | 2 (1.34%) | 1 (0.67%) | 0 (0%) | 0 (0%) | 2 (0.4%) | 2 (0.4%) | 0 (0%) |
| Olanzapine | 0 (0%) | 0 (0%) | 0 (0%) | 0 (0%) | 0 (0%) | 0 (0%) | 0 (0%) | 0 (0%) | 0 (0%) | 0 (0%) | 1 (0.67%) | 0 (0%) | 0 (0%) | 0 (0%) | 1 (0.2%) | 0 (0%) |
| Quetiapine | 0 (0%) | 0 (0%) | 0 (0%) | 1 (0.42%) | 0 (0%) | 0 (0%) | 0 (0%) | 0 (0%) | 0 (0%) | 0 (0%) | 1 (0.67%) | 0 (0%) | 0 (0%) | 0 (0%) | 1 (0.2%) | 1 (0.2%) |
| **Antidepressants total** | 0 (0%) | 3 (1.26%) | 6 (2.51%) | 2 (0.84%) | 0 (0%) | 8 (6.84%) | 3 (2.56%) | 2 (1.71%) | 0 (0%) | 20 (13.42%) | 12 (8.05%) | 1 (0.67%) | 0 (0%) | 31 (6.14%) | 21 (4.16%) | 5 (0.99%) |
| Citalopram | 0 (0%) | 0 (0%) | 0 (0%) | 0 (0%) | 0 (0%) | 0 (0%) | 0 (0%) | 0 (0%) | 0 (0%) | 1 (0.67%) | 0 (0%) | 0 (0%) | 0 (0%) | 1 (0.2%) | 0 (0%) | 0 (0%) |
| Escitalopram | 0 (0%) | 0 (0%) | 1 (0.42%) | 0 (0%) | 0 (0%) | 0 (0%) | 1 (0.85%) | 0 (0%) | 0 (0%) | 2 (1.34%) | 1 (0.67%) | 0 (0%) | 0 (0%) | 2 (0.4%) | 3 (0.59%) | 0 (0%) |
| Fluoxetine HCl | 0 (0%) | 3 (1.26%) | 3 (1.26%) | 2 (0.84%) | 0 (0%) | 6 (5.13%) | 1 (0.85%) | 1 (0.85%) | 0 (0%) | 14 (9.4%) | 3 (2.01%) | 0 (0%) | 0 (0%) | 23 (4.55%) | 7 (1.39%) | 3 (0.59%) |
| Fluvoxamine | 0 (0%) | 0 (0%) | 0 (0%) | 0 (0%) | 0 (0%) | 1 (0.85%) | 0 (0%) | 0 (0%) | 0 (0%) | 1 (0.67%) | 2 (1.34%) | 0 (0%) | 0 (0%) | 2 (0.4%) | 2 (0.4%) | 0 (0%) |
| Mirtazapine | 0 (0%) | 0 (0%) | 1 (0.42%) | 0 (0%) | 0 (0%) | 0 (0%) | 0 (0%) | 0 (0%) | 0 (0%) | 0 (0%) | 1 (0.67%) | 0 (0%) | 0 (0%) | 0 (0%) | 2 (0.4%) | 0 (0%) |
| Sertraline | 0 (0%) | 0 (0%) | 1 (0.42%) | 0 (0%) | 0 (0%) | 1 (0.85%) | 1 (0.85%) | 1 (0.85%) | 0 (0%) | 2 (1.34%) | 5 (3.36%) | 1 (0.67%) | 0 (0%) | 3 (0.59%) | 7 (1.39%) | 2 (0.4%) |
| **Melatonin** | 3 (1.26%) | 32 (13.39%) | 14 (5.86%) | 0 (0%) | 4 (3.42%) | 16 (13.68%) | 5 (4.27%) | 1 (0.85%) | 3 (2.01%) | 33 (22.15%) | 10 (6.71%) | 1 (0.67%) | 10 (1.98%) | 81 (16.04%) | 29 (5.74%) | 2 (0.4%) |
| **Polypharmacy ≥2  including melatonin** | 3 (1.26%) | 53 (22.18%) | 34 (14.23%) | 2 (0.84%) | 2 (1.71%) | 14 (11.97%) | 4 (3.42%) | 1 (0.85%) | 2 (1.34%) | 51 (34.23%) | 23 (15.44%) | 1 (0.67%) | 7 (1.39%) | 118 (23.37%) | 61 (12.08%) | 4 (0.79%) |

*Note:* Percentages were calculated with respect to total *n* within each diagnostic group, e.g. , e.g. *n* = 239 for ADHD group, *N* = 505 for the total sample. *Polypharmacy ≥2 including melatonin* = concurrent use of two or more medications, including melatonin.
